# Therapeutic Targeting of Fibroblast Growth Factor Receptors in Gastric Cancer

**DOI:** 10.1155/2015/796380

**Published:** 2015-04-27

**Authors:** Mikito Inokuchi, Yoshitaka Fujimori, Sho Otsuki, Yuya Sato, Masatoshi Nakagawa, Kazuyuki Kojima

**Affiliations:** ^1^Department of Surgical Oncology, Tokyo Medical and Dental University, 1-5-45, Yushima, Bunkyo-ku, Tokyo 113-8519, Japan; ^2^Department of Minimally Invasive Surgery, Tokyo Medical and Dental University, 1-5-45, Yushima, Bunkyo-ku, Tokyo 113-8519, Japan

## Abstract

Chemotherapy has become the global standard treatment for patients with metastatic or unresectable gastric cancer (GC), although outcomes remain unfavorable. Many molecular-targeted therapies inhibiting signaling pathways of various tyrosine kinase receptors have been developed, and monoclonal antibodies targeting human epidermal growth factor receptor 2 (HER2) have become standard therapy for HER2-positive GC. An inhibitor of vascular endothelial growth factor receptor 2 or MET has also produced promising results in patients with GC. Fibroblast growth factor receptors (FGFR) play key roles in tumor growth via activated signaling pathways in GC. Genomic amplification of FGFR2 leads to the aberrant activation found in GC tumors and is related to survival in patients with GC. This review discusses the clinical relevance of FGFR in GC and examines FGFR as a potential therapeutic target in patients with GC. Preclinical studies in animal models suggest that multitargeted tyrosine kinase inhibitors (TKIs), including FGFR inhibitor, suppress tumor cell proliferation and delay tumor progression. Several TKIs are now being evaluated in clinical trials as treatment for metastatic or unresectable GC harboring FGFR2 amplification.

## 1. Introduction

Gastric cancer (GC) is the second leading cause of cancer-related mortality, with 738,000 deaths per year [1]. Median overall survival was only 10 to 13 months in patients with metastatic or unresectable GC who received combined chemotherapy with cytotoxic agents [2–4].

Aberrant or oncogenic activation of receptor tyrosine kinase (RTK) is involved in carcinogenesis or tumor progression. Inhibition of signaling pathways of RTK is most intensively pursued as an anticancer target. Trastuzumab, a monoclonal antibody against human epidermal growth factor receptor 2 (HER2/ERBB2), was the first RTK-targeting agent approved for the indication of unresectable or metastatic GC worldwide [5]. However, several agents targeting epidermal growth factor receptor (EGFR) provided no additional benefits in clinical trials [6–8]. Bevacizumab, a monoclonal antibody targeting vascular endothelial growth factor- (VEGF-) A, which activates VEGF receptor- (VEGFR-) 1 and VEGFR-2, provided significant benefits in terms of progression-free survival (PFS), but not overall survival (OS) [9]. Ramucirumab is a monoclonal antibody targeting the extracellular domain of VEGFR-2. Ramucirumab as second-line chemotherapy prolonged overall survival [10, 11] and was recently approved for the indication of unresectable or metastatic GC. Rilotumumab is a monoclonal antibody designed to inhibit binding of HGF to c-MET. Its additive effect was clinically significant in GC with high c-MET expression [12].

Fibroblast growth factor receptors (FGFRs) are one of the RTK families that belong to the immunoglobulin (Ig) superfamily [13]. Binding of fibroblast growth factors (FGFs) with high-affinity to FGFR results in kinase activation of downstream signaling pathways. The FGFR family consists of 5 receptors, named FGFR1 to FGFR5. The extracellular regions of FGFRs comprise 3 extracellular Ig-like domains (I–III), a single transmembrane domain, and the cytoplasmic tyrosine kinase domains TK1 and TK2. However, FGFR5 lacks an intracellular tyrosine kinase domain. The extracellular Ig-II and Ig-III domains are the FGF ligand-binding sites. Alternative splicing of Ig-III occurs in FGFRs 1–3, creating IIIb and IIIc variants of the receptors with diverse ligand-binding specificities that are expressed in a tissue-specific manner [14–16]. Binding of FGFs to FGFRs induces receptor dimerization, conformational changes within the FGFR structure, and phosphorylation of tyrosines in the intracellular part of the receptor, including the kinase domain and the C-terminus [17]. Subsequent downstream signaling is activated in two main pathways via the intracellular receptor substrates FGFR substrate 2 (FRS2) and phospholipase Cg, leading ultimately to upregulation of the Ras-dependent mitogen-activated protein kinase (MAPK)/extracellular signal-regulated kinase (ERK) and Ras-independent phosphoinositide 3-kinase (PI3K)/Akt signaling pathways [18]. The other signaling pathway, dependent on signal transducer and activator of transcription (STAT), is activated by FGFRs [14].

## 2. Clinical Analysis of Expression or Genomic Alteration of FGFR in GC

The results of immunohistochemical analyses of FGFRs are summarized in Table 1. We previously showed that protein overexpression of FGFR1, FGFR2, and FGFR4 is significantly associated with tumor depth, lymph-node metastasis, tumor stage, and poorer survival in GC, while FGFR3 is not [19]. Others have shown that overexpression of K-sam, a FGFR2 homologue, is significantly related to pathologically undifferentiated or diffuse-type GC [20, 21]. Nagatsuma et al. reported that FGFR2 overexpression is significantly associated with tumor depth, lymph-node metastasis, and tumor stage in a larger analysis [22]. Moreover, patients with FGFR2 overexpression had a significantly higher incidence of peritoneal or lymph-node recurrence and a significantly shorter survival than those without FGFR2 overexpression. Ye et al. showed that FGFR4 is not associated with any clinicopathological factors or with survival, although patients with far advanced GC and FGFR4 overexpression had significantly worse survival [23]. The mRNA expression of* FGFR1*,* FGFR2*, or* FGFR4* was upregulated in GC as compared with that in normal tissues, although* FGFR3* mRNA was barely detectable in normal as well as cancer tissue [24].

Studies of* FGFR* genomic alterations are summarized in Table 2.* FGFR2* amplification is a well-known phenomenon in GC. The frequency of* FGFR2* amplification on comparative genomic hybridization had been reported to be 7% (2 of 30) in GC in one study and 16% (3 of 19) in diffuse-type GC in another [25, 26]. In a study using Southern blot analysis, the frequency of* FGFR2* amplification was 5% (3 of 57) [27]. Betts et al. reported that* FGFR2* amplification was detected on fluorescence in situ hybridization (FISH) analysis in 1.8% (3 of 171) of GCs, and survival was very poor in three patients who had tumors with* FGFR2*-amplification [28]. In a study by Jung et al.,* FGFR2* amplification was detected on FISH in 4.5% (14 of 313) of GCs and was significantly associated with the depth of tumor invasion, lymph-node metastasis, distant metastasis, tumor stage, and poorer survival [29]. In that study,* FGFR2* amplification was not detected in papillary or well-differentiated subtypes of GC. Das et al. reported that* FGFR2* amplification was found in 7.3% (10 of 137) of patients, while* FGFR2* deletion was detected in 5.8% (8 of 137), and patients with* FGFR2*-amplified GC had worse survival than those with* FGFR2*-deleted GC [30]. Interestingly, they showed that not only* FGFR2* amplification but also deletion was more common in undifferentiated type than in differentiated type. In an international multicenter study using FISH, the presence of* FGFR2* amplification did not differ appreciably among three countries: 7.4% (30 of 408) in the UK, 4.6% (9 of 197) in China, and 4.2% (15 of 356) in Korea [31]. In each country, patients with* FGFR2*-amplified GCs had worse survival than those with nonamplified GCs. In addition, 24.1% of* FGFR2*-amplified GCs displayed intratumoral heterogeneity within multiple samples extracted from the same tumors on tissue microarray analysis. In the FISH studies mentioned above,* FGFR2* gene amplification was determined on the basis of the presence of tight signals of* FGFR2* clusters or a ratio of* FGFR2* signals to chromosome enumeration probe-10 signals of 2.0 or higher.

Matsumoto et al. reported that* FGFR2* amplification on copy number assay (more than 5 copies) was detected in 4.1% (11 of 267) of GCs, whereas amplification of other* FGFRs* was not detected [32]. Patients with* FGFR2*-amplified tumors had slightly but not significantly shorter survival than those with nonamplified tumors. In a comprehensive survey assessing genomic alterations of GCs by high-resolution single nucleotide polymorphism arrays,* FGFR2* amplification was detected in 9.3% (18 of 193) of GCs, and coamplification of* FGFR2* with* EGFR*,* ERBB2*,* KRAS*, or* MET* was rarely detected [33]. In that study, the overall survival of patients with* FGFR2* amplification did not differ from that of patients with nonamplification, although the survival of patients with high mRNA expression of* FGFR2* was significantly worse than that of patients with low mRNA expression of* FGFR2* in the extended population. Wang et al. detected* FGFR2* amplification in 3.0% (3 of 100) of GCs on single nucleotide polymorphism (SNP) genotyping arrays [34]. In addition, two mutations of* FGFR2* were identified in GC: a missense in exon IIIa and a splice site mutation in exon IIIc [35].

Among other* FGFR *genes, Ye et al. investigated the SNP of* FGFR4* (Gly388 to Arg388) in GC samples and showed that 45% (46 of 103) of patients were heterozygous and 13% (13 of 103) homozygous for Arg388 allele [36]. Patients with tumors in which* FGFR4* Arg388 allele was found had significantly shorter survival, and the presence of* FGFR4* Gly388Arg allele was an independent prognostic factor.* FGFR1* somatic mutation on whole-exome sequencing was detected in 1.1% (1 of 87) of diffuse type GCs and in 3.9% (2 of 51) of intestinal type GCs [37].

## 3. Preclinical Studies of FGFR Inhibition in GC Cells

*FGFR2*-amplified GC cell lines have high expression of FGFR2 protein or* FGFR2* mRNA [32, 38]. On the other hand, the promoter region of* FGFR2 *gene is highly methylated, and* FGFR2* mRNA expression is markedly reduced in several GC cell lines (SNU-1, SNU-5, SNU-484, and SNU-638) [39].* FGFR2* mRNA expression was restorable by demethylation using 5-aza-2′-deoxycytidine in cell lines with methylation of the promoter region of FGFR2, suggesting that aberrant hypermethylation of* FGFR2* gene might lead to loss of FGFR2 expression.

Zhao et al. generated two effective monoclonal antibodies that recognize different epitopes on FGFR2: GAL-FR21, binding to only IIIb isoform of FGFR2, and GAL-FR22, binding to both IIIb and IIIc isoforms [40]. GAL-FR21 and GAL-FR22 blocked the binding of FGFs to FGFR2 IIIb, and GAL-FR21 inhibited FGF-induced phosphorylation of FGFR2. Both antibodies downregulated FGFR2 expression on SNU-16, an* FGFR2*-amplified GC cell line and effectively inhibited the growth of SNU-16 xenograft tumors.

GP369 is an FGFR2-IIIb-specific antibody and blocked phosphorylation of FGFR2, FRS2 tyrosine, and ERK in a GC cell line (SNU-16) overexpressing FGFR2-IIIb [41]. GP369 treatment potently inhibited the growth of SNU-16 xenograft tumors.

Small-molecule compounds fitting into the ATP-binding pockets of RTKs have been developed as anticancer drugs. PD173074 is a reversible inhibitor of FGFR and VEGFR. PD173074 blocks FGF2-induced angiogenesis [42] and also blocks mitogenesis of tumor cells via G1-arrest mediated by downregulation of cyclin D1 and cyclin D2 [43]. Treatment with PD173074 selectively and potently inhibited growth of* FGFR2*-amplified GC cell lines (KATOIII, SNU-16, and OCUM-2M), leading to a strong decrease in tumor cells in S phase accompanied by an increase in tumor cells in the sub-G1 population [38]. In addition, prominent induction of poly(ADP-ribose) polymerase, a marker of caspase activation associated with apoptosis, was observed after treatment. EGFR family kinases might have been downstream targets of amplified* FGFR2* in that study, because the increased expression of phosphorylated HER receptors was dependent on FGFR2. PD173074 was more effective in* FGFR2*-amplified GC cell lines (SNU-16, TU-KATOIII, HSC-43, and HSC-39) than in nonamplified cell lines (OCUM1, IM95, 58Aa1, and 44As3) on growth inhibition assays [32].

Ki23057, a small-molecule-acting FGFR and VEGFR autophosphorylation inhibitor, significantly suppressed the proliferation of scirrhous cancer cells (OCUM-2MD3 and OCUM-8), but not nonscirrhous cancer cells (MKN-7, MKN-45, and MKN-74) [44]. Administration of Ki23057 prolonged survival in a mouse model of peritoneal dissemination prepared using OCUM-2MD3. Ki23057 mainly inhibited the downstream RAS-ERK signaling pathway rather than another PI3K-Akt pathway.

Cediranib (AZD2171) is also a broad-range tyrosine kinase inhibitor (TKI) and inhibits FGFR, VEGFR, PDGFR, and KIT, as well as VEGF-induced proliferation of human endothelial cells [45]. Cediranib completely inhibited the phosphorylation of FGFR2 and downstream targets, including FRS2, Akt, and MAPK, in GC cell lines (KATO-III and OCUM2M) that strongly expressed FGFR2-IIIb mRNA, and then significantly and dose-dependently inhibited tumor growth in KATO-III and OCUM2M tumor xenografts [46].

AZD4547 is a highly selective and potent ATP-competitive TKI of FGFR1–3 and inhibited recombinant FGFR kinase activity in vitro and suppressed FGFR signaling and growth in tumor cell lines with deregulated FGFR expression [47]. After treatment of GC cell-lines (SNU-16 and KATO III) with AZD4547, expression levels of phosphorylated FGFR2 and its downstream signaling molecules, such as phospholipase C-gamma, FRS2, ERK, and S6, were all reduced [48]. Furthermore, treatment with AZD4547 also dose-dependently increased the sub-G1 population of GC cells. AZD4547 inhibited tumor regression in* FGFR2*-amplified xenografts (SNU-16) but not in nonamplified models (AZ521 and MGC803) in that study. In addition, antitumor efficacy was enhanced in vivo by combined chemotherapy with AZD4547 plus chemotherapeutic agents as compared with monotherapy.

Ponatinib (AP24534) was designed with a carbon-carbon triple bond to accommodate the T315I mutation in the ABL kinase domain [49]. Ponatinib potently inhibits the kinase activity of FGFR1–4 and had higher inhibitory activity in GC cells with FGFR2 amplification than did other FGFR inhibitors and inhibited the growth of SNU-16 xenograft tumors [50]. In addition, ponatinib potently inhibited cell proliferation and signaling in several cell lines of other cancers with FGFR mutation.

S49076, a potent inhibitor of FGFR1–3, MET, and AXL, inhibited the autophosphorylation of those receptors and the phosphorylation of FRS2 [51]. S49076 inhibited viability in SNU-16 cell lines and tumor growth in SNU-16 xenografts. Combined treatment with S49076 and bevacizumab, a VEGF inhibitor, enhanced the antitumor effect in other cancer xenografts.

Dovitinib (TKI258) is an oral multitargeted TKI of FGFR1–3, VEGFR, platelet-derived growth factor receptor (PDGFR), FMS-like tyrosine kinase 3 (FLT-3), KIT, and colony stimulating factor 1. The potent growth inhibitory activity of dovitinib was specifically observed in* FGFR2*-amplified GC cell lines (KATO-III and SNU-16) [33]. Dovitinib treatment decreased phosphorylation of FGFR2, Akt, and ERK and inhibited soft agar colony formation in* FGFR2*-amplified GC cell lines, although additional factors might be required to induce apoptosis by dovitinib treatment. Dovitinib inhibited tumor growth in an* FGFR2*-amplified primary human GC xenograft model [33].

Small interfering RNA (siRNA), the intermediate product of the pathway of RNA interference, plays a key role in RNA silencing treatment. Silencing of* FGFR* expression by treatment with siRNA led to inhibition of proliferation and promotion of apoptosis accompanied by a reduction in VEGFR expression and a rise in caspase-3, an apoptosis-related protein, in an in vitro study [52]. In experimental in vivo studies using GC cells (MGC80-3), siRNA also suppressed the expression of FGFR and enhanced tumor shrinkage [52].

MicroRNAs (miR) negatively regulate protein expression by binding to protein-coding mRNAs and inhibiting translation. The 3′UTR of* FGFR1* mRNA contains two putative binding sites of miR-133b [53]. Therefore, miR-133b reduced the protein expression of FGFR1. Furthermore, upregulation of FGFR1 expression was found to negatively correlate with miR-133b expression in several GC lines and GC tissues.

## 4. Clinical Trials of FGFR-Targeted Treatment in GC

Clinical trials of FGFR inhibitors for GC are summarized in Table 3. Several phase II trials of FGFR inhibitors are ongoing in GC. Dovitinib was evaluated in a phase I study of 35 solid tumors including 2 GCs [54]. Enrolled patients were treated in four intermittent (25–100 mg/day) and three continuous (100–175 mg/day) dosing cohorts. Dose-limiting toxicities were grade 3 hypertension in one patient in the 100 mg continuous dosing cohort, grade 3 anorexia in a second patient at 175 mg, and grade 3 alkaline phosphatase elevation in a third patient at 175 mg. Unfortunately, neither patient with GC had stable disease for more than 4 months in this study. Nonetheless, three phase II studies of dovitinib are ongoing in GC. Dovitinib is being assessed as salvage monotherapy after failure of first- or second-line chemotherapy in patients with advanced or metastatic scirrhous GC in one study [55] and in patients with GC harboring* FGFR2* amplification in another study [56]. Dovitinib was administered orally at 500 mg/day on days 1 to 5 of 7-day repeated cycles in both studies. In the third study, divided into phase I and phase II, dovitinib is being assessed in combination with docetaxel as second-line chemotherapy in patients with GC [57].

A phase II study of AZD4547, an oral TKI of both FGFR and VEGFR, is also ongoing to assess the efficacy and safety of AZD4547 monotherapy versus paclitaxel in patients with locally advanced or metastatic GC associated with* FGFR2* polysomy or amplification [58]. AZD4547 was administered orally at 160 mg/patients on days 1 to 14 of a 21-day cycle.

## 5. Results of Clinical Trials of FGFR-Targeted Treatment in Various Cancers

Phase III clinical trials in patients with other types of cancer are shown in Table 4. Cediranib (AZD2171) is an oral TKI of both FGFR and VEGFR. In one study of colorectal cancer, the addition of cediranib to standard first-line chemotherapy significantly prolonged PFS but not OS [59]. In the other study, the noninferiority of cediranib did not reach the predefined level of PFS as compared with bevacizumab [60]. No synergistic effect of cediranib was found in patients with non-small-cell lung carcinoma (NSCLC) [61]. Brivanib (BMS-582664) is an oral TKI of both FGFR and VEGFR, and the addition of brivanib increased toxicity and did not improve OS as compared with cetuximab alone in patients with colorectal cancer with wild-type KRAS [62]. In addition, no significant effect of brivanib was found in unresectable hepatocellular carcinoma [63, 64]. Dovitinib (TKI258) is an oral multitargeted TKI, including FGFR, and was not superior to sorafenib in metastatic renal cell carcinoma [65]. Nintedanib (BIBF1120) is an oral TKI of FGFR, VEGFR, PDGFR, FLT-3, and lymphocyte-specific protein tyrosine kinase and significantly prolonged PFS in combination with docetaxel in patients with NSCLC [66].

## 6. Conclusions

Aberrant activation of FGFR signaling pathway, especially* FGFR2* amplification, is related to disease progression or poor survival in GC; thus FGFR-targeted therapy is considered promising. Unfortunately, the superiority of multitargeted TKIs, including those with FGFR inhibitory activity, to standard chemotherapy has not been demonstrated in most phase III clinical trials in other malignancies. However, TKIs were evaluated as VEGFR inhibitors, but not FGFR inhibitors, in those studies. FGFR inhibitors were shown to have higher antitumor activity against* FGFR2*-amplified tumors than against nonamplified tumors in preclinical studies [32, 33, 38, 40–42, 46, 48, 50, 51]. Therefore, ongoing clinical trials of dovitinib or AZD4547 in patients with* FGFR2*-amplified GC are expected to show positive results. Scirrhous gastric cancer is known to be refractory to intensive treatment and to carry a poor prognosis; however,* FGFR2* amplification is found in cell lines originating from scirrhous GC, such as KATO-III, SNU-16, and OCUM-2M. FGFR inhibitors may be a promising treatment for scirrhous GC and are now being evaluated in clinical trials. On the other hand, intratumoral heterogeneity of* FGFR2* amplification has been found in GC samples [32]. Intratumoral heterogeneity of HER2 was also detected in GC, and the expression levels of primary lesions may not be consistent with those of metastatic sites. Intratumoral heterogeneity can be a critical issue for a single molecular-targeted treatment [67].

Amplification of other FGFRs has not been found in GC; however, overexpression of FGFR1 and FGFR4 or single nucleotide polymorphism of* FGFR4* appears to be associated with tumor progression or survival [19, 23, 36]. Preclinical studies evaluating other FGFRs in GC remain scant.* FGFR2 *amplification was detected in only 1.8% to 7.3% of patients with GC, regardless of ethnic group; therefore, only a small subgroup of patients with GC can potentially benefit from FGFR2-targeted therapy alone. The development of FGFR inhibitors against tumors with overexpression not only of FGFR2 but also of FGFR1 or FGFR4 is likely to enhance potential treatment benefits in patients with GC.

## Figures and Tables

**Table 1:** FGFR protein expressions on immunohistochemical analysis and clinical outcomes in GC.

	*n*	Definition of positivity	Positive case %	Relation to clinicopathological factors	Relation to survival	Reference
FGFR1	222	Scoring system of intensity + extensity	29	T, N, M, stage	Worse	[19]

FGFR2	950	2+ or 3+, >50%	31	T, N, M, stage	Worse	[22]
	222	Scoring system of intensity + extensity	51	T, N, M, stage	Worse	[19]
	136	Stronger than normal epithelium	31	T, peritoneal Dissemination, diffuse type	Worse	[20]
	49	Stronger than normal epithelium	41	Stage Undifferentiated type	Worse	[21]

FGFR3	222	Scoring system of intensity + extensity	64	NA	NA	[19]

FGFR4	222	Scoring system of intensity + extensity score	79	T, N, M, stage	Worse	[19]
	94	3+, >10%	38	NA	Worse	[23]

T: tumor depth; N: lymph-node metastasis; M: distant metastasis; NA: not assisted.

**Table 2:** *FGFR* gene alterations in GC.

	Method	*n*	Positive expression definition	%	Relation to clinicopathological factors	Relation to survival	Reference
*FGFR2* amplification	FISH	961	*FGFR2/CEP-10* ratio ≥2 or *FGFR2* gene clusters in ≥10%	5.6	N	Worse	[31]
	FISH	313	*FGFR2/CEP-10* ratio ≥2 or *FGFR2* gene clusters	4.5	T, N, M, stage	Worse	[29]
	FISH	171	*FGFR2/CEP-10* ratio ≥2	1.8	ND	Worse	[28]
	FISH	137	*FGFR2/CEP-10* ratio ≥2	7.3	Undifferentiated type	Worse	[30]
	RT-PCR	267	*FGFR2* >5 copies	4.1	NA	worse	[32]
	SNP microarray	193	GISTIC algorithm	9.3	NA	NA	[33]
	SNP microarray	100	GISTIC algorithm	3.0	ND	Not investigated	[34]
*FGFR4* SNIP	PCR-RFLP	103	Arg388 allele	57	NA	Worse	[36]
*FGFR1* mutation	Whole-exome sequence	138		2.2	ND	ND	[37]

FISH: fluorescence in situ hybridization; RT-PCR: reverse-transcription polymerase chain reaction; SNP: single nucleotide polymorphism; PCR-RFLP: polymerase chain reaction-restriction fragment length polymorphism analysis; CEP: chromosome enumeration probe; GISTIC: the genomic identification of significant targets in cancer; T: tumor depth; N: lymph-node metastasis; M: distant metastasis; NA: not assisted; ND: not described.

**Table 3:** Clinical trials of FGFR-targeting agents in GC.

Agent	Target	Type of cancer	Phase	Combined regimen	Status	Reference
Dovitinib (TKI258)	FGFR, VEGFR, PDGFR, FLT-3, KIT, and CSF-1	Gastric (scirrhous type)	II	None	Ongoing	[55]
		Gastric (*FGFR2* amplification)	II	None	Ongoing	[56]

AZD4547	FGFR and VEGFR	Gastric	II	Docetaxel	Ongoing	[57]
		Gastric (*FGFR2* amplification)	II	Paclitaxel	Ongoing	[58]

**Table 4:** Phase III clinical trials of FGFR-targeting agents.

Agent	Target	Type of cancer	Phase	Combined regimen (comparative arm)	Status or result	Reference
Cediranib (AZD2171)	FGFR and VEGFR	CRC	III	FOLFOX or CAPOX (FOLFOX or CAPOX + placebo)	Negative on OS	[59]
		CRC	III	FOLFOX (FOLFOX + bevacizumab)	Negative on PFS	[60]
		NSLSC	III	Carboplatin + paclitaxel (carboplatin + paclitaxel + placebo)	Negative on PFS/OS	[61]

Brivanib (BMS582664)	FGFR and VEGFR	CRC (wild-type *KRAS*)	III	Cetuximab (cetuximab + placebo)	Negative on OS	[62]
		HCC	III	None (sorafenib)	Negative on OS	[63]
		HCC	III	None (placebo)	Negative on OS	[64]

Dovitinib (TKI258)	FGFR, VEGFR, PDGFR, FLT-3, KIT, and CSF-1	RCC	III	None (sorafenib)	Negative on PFS	[65]

Nintedanib (BIBF1120)	FGFR, VEGFR, PDGFR, FLT-3, and LCK	NSCLC	III	Docetaxel (docetaxel + placebo)	Positive on PFS	[66]

Lenvatinib (E7080)	FGFR, VEGFR, and PDGFR	HCC	III	None (sorafenib)	Ongoing	[68]
		Thyroid	III	None (placebo)	Ongoing	[69]

Orantinib (TSU68)	FGFR, VEGFR, and PDGFR	HCC	III	None (placebo)	Suspended	[70]

CRC: colorectal cancer; NSCLC: non-small-cell lung cancer; HCC: hepatocellular carcinoma; RCC: renal cell carcinoma; FOLFOX: 5-fluorouracil + leucovorin + oxaliplatin; CAPOX: capecitabine + leucovorin + oxaliplatin; RFS: relapse-free survival; OS: overall survival.
